# Folate status in women of childbearing age in the Urban Metropolitan Region of Chile: results from the National Health Survey 2016–2017

**DOI:** 10.1017/S1368980020002608

**Published:** 2021-02

**Authors:** Dolores Busso, Guadalupe Echeverría, Alvaro Passi-Solar, Fernanda Morales, Marcelo Farías, Paula Margozzini

**Affiliations:** 1 Universidad de los Andes, Chile, School of Medicine, Biomedical Research and Innovation Center, Monseñor Álvarez del Portillo 12455, 7620001 Las Condes, Santiago, Chile; 2 Pontificia Universidad Católica de Chile, School of Medicine, Department of Nutrition, Diabetes and Metabolism, Santiago, Chile; 3 Pontificia Universidad Católica de Chile, School of Medicine, Center of Molecular Nutrition and Chronic Diseases, Santiago, Chile; 4 Pontificia Universidad Católica de Chile, School of Medicine, Department of Public Health, Diagonal Paraguay 362, 8330077 Santiago, Chile; 5 University College London, Department of Epidemiology and Public Health, London, UK; 6 Pontificia Universidad Católica de Chile, School of Medicine, Division of Obstetrics and Gynecaology, Santiago, Chile

**Keywords:** Folic acid fortification, Plasma folate levels, Neural tube defects, Reproductive age, Chile

## Abstract

**Objective:**

To analyse serum folate levels in women of childbearing age in the Metropolitan Region (MR) of Chile.

**Design:**

Cross-sectional design as part of the 2016–2017 National Health Survey (*Encuesta Nacional de Salud, ENS 2016–2017*), using a household-based multistage stratified random sample. Serum folate levels measured by electrochemiluminescence immunoassay in fasting venous blood samples were classified as deficient (<4·4 ng/ml), normal (4·4–20 ng/ml) or supraphysiological (>20 ng/ml).

**Setting:**

The MR of Chile.

**Participants:**

Women of reproductive age (15–49 years, *n* 222) from the MR participated in the ENS 2016–2017.

**Results:**

The mean, median and range of serum folate were 14·2 (se 0·4), 13·9 and 2·1–32·2 ng/ml, respectively. Folate deficiency was detected in 0·9 % of women, while 7·0 % had supraphysiological levels of the vitamin. No significant effects of age, educational level, marital status, parity, smoking status or nutritional status on serum folate levels were detected by univariate or multivariate analyses. Intake of folic acid supplements showed a significant association with serum folate levels, but only 1·2 % of women used supplements.

**Conclusions:**

Folate deficiency in women of reproductive age living in the MR of Chile is almost inexistent according to the ENS 2016–2017, suggesting that the current population-wide mandatory folic acid fortification of flour is an effective and equitable measure to prevent folate deficiency. These results support the option of maintaining current folic acid fortification in Chile, particularly based on the low adherence to supplementation regimes evidenced in other populations.

In 1991, the UK Medical Research Council demonstrated that periconceptional supplementation with folic acid could significantly reduce the incidence of neural tube defects (NTD)^(1)^. NTD are a group of congenital malformations resulting from incomplete closure of the neural tube, the embryonic precursor of the brain and spinal cord^(2)^. Because human neural tube closure takes place 3 weeks after conception, before most women are even aware of their pregnancy^(3)^, adequate folate levels should be provided to all fertile women to be effective at preventing NTD. This situation is particularly relevant in regions such as Latin America, where 69 % of pregnancies are unintended^(4)^.

Since the early 1990s, different strategies to increase blood folate concentration in women of reproductive age have been considered. Interventions designed to increase the intake of folate-rich foods were rapidly discarded, as they were ineffective in improving folate status^(5)^. In most populations, folic acid supplement use is also ineffective because of low awareness of patients and healthcare providers to the use of folic acid before pregnancy^(6,7)^, together with low adherence to the use of pills and with the existence of socio-economic disparities (high costs and low availability) of vitamins in specific locations^(8,9)^. Folic acid mandatory fortification of food is considered an effective measure to provide sufficient folate to women before they get pregnant. Enrichment of cereal grain products with folic acid was first implemented in the USA in 1998^(3)^. Since then, folic acid fortification of wheat, maize and/or rice flour has been adopted as a cost-effective public health policy in more than eighty countries^(10,11)^, and its implementation has proven to reduce the incidence of NTD significantly^(12,13)^.

In Chile, mandatory fortification of wheat flour with 2·2 mg folic acid/kg was first implemented in 2000. This policy was designed to increase the average intake of folic acid by 400 µg/d in women of reproductive age. In 2003, Hertrampf *et al*.^(14)^ showed that serum folate levels had risen from 4·3 to 16·4 ng/ml in a cohort of 605 young women from low socio-economic households. The policy showed to be effective in decreasing the incidence of NTD by 43 % from pre-fortification (1999–2000) to post-fortification (2001–2002)^(15)^. The cost-effectiveness of this intervention was later demonstrated by Llanos *et al*.^(16)^, who showed that the overall cost of surgery and long-term rehabilitation for patients with NTD ‘significantly exceeded the investment incurred by the milling industry’.

Evidence worldwide and from Chile suggested that specific population subgroups or individuals might suffer adverse effects if exposed to high levels of folic acid^(17–21)^. Positive associations between high serum folate with anaemia and enhanced cognitive decline in the elderly with vitamin B_12_ deficiency, as well as increased risk or progression of some types of cancer, were shown both internationally and in Chile^(20–24)^. High folic acid intake was also shown to interfere with the action of certain drugs, for example, those used to treat epilepsy, some autoimmune diseases and malaria^(17)^. Those potential harmful effects of high folic acid intake, together with international concerns about the increased supply of foods fortified with folic acid, led the Chilean government to adjust the fortification policy in 2009 to 1·8 mg/kg wheat flour (range 1·0–2·6 mg/kg).

In this work, we analysed the current folate status of a subgroup of Chilean women of childbearing age for the first time after the implementation of the policy adjustment. The current study, aimed at providing evidence to assess the need for adjustment of the public policy of flour fortification with folic acid, was part of the 2016–2017 National Health Survey (Encuesta Nacional de Salud, ENS 2016–2017).

## Methods

### Participants and data collection

All women of childbearing age participating in the ENS 2016–2017 and living in the Metropolitan Region (MR) were selected for folate determinations, because of budget and logistic constraints (further information in the last paragraph of this section). The MR is one of the sixteen administrative and political regions from Chile. Although it is one of the smallest regions, it is the most densely populated and contains the capital city (Santiago) and its urban suburbs.

**Fig. 1:**
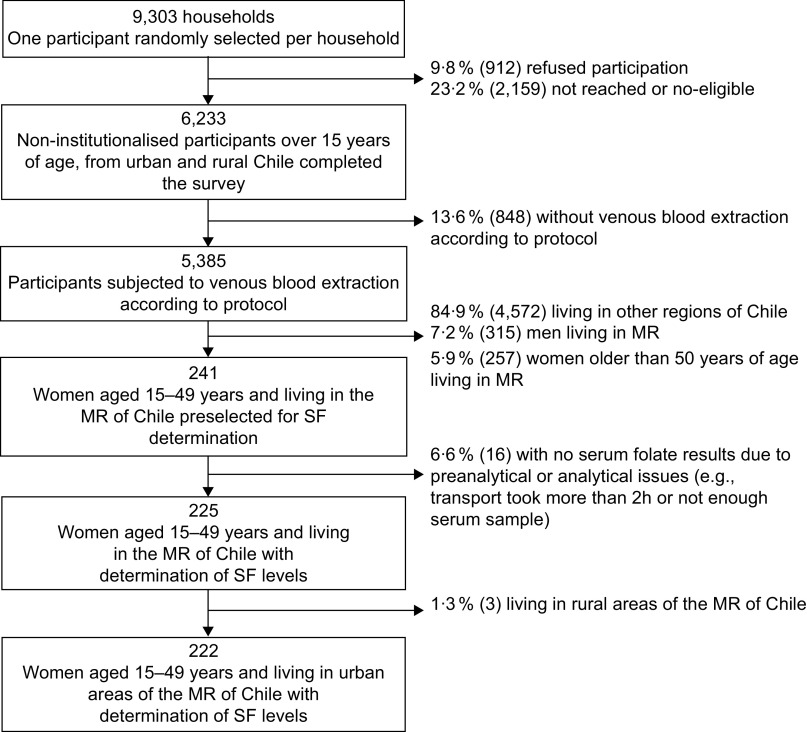
Flow diagram of the subsample selected from Encuesta Nacional de Salud 2016–2017 for serum folate (SF) determinations: women of childbearing age living in the urban Metropolitan Region (MR) of Chile

The ENS 2016–2017 is a national cross-sectional study that used stratified, multistage and clustered random sampling of households. The strata included fifteen administrative regions and urban/rural areas, from where counties were selected according to the proportion of people aged 15 years or more. Households and one person per home were selected at random in each county. The ENS was designed to estimate, at the national and regional levels, the prevalence of common chronic conditions (e.g., expected 6 and 30 % of diabetes and hypertension, respectively) and associated risk factors by sex and age groups. The Kish method was used to select one participant per household randomly^(25)^. The number of selected households was 9303: 23·2 % were not present at home (not reached) or were non-eligible (i.e., pregnant women or persons showing aggressive behaviour, with potential risk for the interviewer) and 9·8 % refused to participate. The household national survey response rate (participants interviewed/participants sampled) was 67 %^(26)^. No replacements were performed. The participants included in the ENS 2016–2017 survey were 6233 non-institutionalised men and women aged 15 years or more (Fig. 1). In the MR, the response rate at the first visit was 62 % (31 and 7 % non-contact and refusal, respectively). The levels of response both nationwide and in the MR are comparable to those achieved by other national health examination surveys^(26)^.

Data were collected in two visits, lasting nearly 1 h each. During the first home visit, a lay interviewer applied health questionnaires using electronic data capture devices. Eighty-nine percentage of the interviewed participants received a second visit by a trained nurse, who applied questionnaires, measured blood pressure, conducted anthropometric measurements and collected fasting blood and urine samples. The nurse also recorded the medications participants were currently using (prescribed or not) via a detailed inventory. Medications were classified using the anatomical therapeutic chemical (ATC) classification system, in which supplements of folate and derivatives are given the code ATC B03BB (i.e., Ref. (27)). Similar response rates to the nurse visit were registered in the MR and the rest of the country.

Weight assessment was done with the HN289 OMRON digital scale (OMRON Healthcare Co. Ltd, accuracy 5–150 kg: ± (1 % + 0·1 kg)). Height was measured with a set square and a chrome-plated tape measure against a wall, as reported previously^(28,29)^. The maximum period of stability of folate samples protected from light, if refrigerated, is 2 d^(30)^. Among 241 women in childbearing age in the MR, 225 blood samples were processed to obtain serum within 1 h of their collection and transported in <2 d at 4°C to the Clinical Laboratory Red Salud UC CHRISTUS from Pontificia Universidad Católica de Chile in Santiago. Women subjected to serum folate analyses from the MR were mainly from urban areas (*n* 222), and only three women lived in rural areas, so the latter were excluded from the analyses.

### Determination of serum folate concentrations

Serum folate concentrations were measured by electrochemiluminescence immunoassay, using the Roche Diagnostics ‘Cobas 8000-E602 Immunoassay Analyzer’. The assay coefficients of variation were 10·6 and 7·3 % at mean iodine concentrations of 2·5 and 6·7 ng/ml, respectively.

The serum folate levels were classified into deficient (<4·4 ng/ml), normal (4·41–20 ng/ml) or supraphysiological (>20 ng/ml) according to the WHO’s guidelines^(31)^. The supraphysiological category was subdivided into two sub-categories: high (>20–26 ng/ml) and very high (>26 ng/ml), according to the reported cut-offs at which free folic acid is detected and cognitive deficits are reported, respectively^(22,32)^.

### Socio-demographic, lifestyle and nutritional status population variables

The following variables were considered in the analysis: age, educational level (low, <8 years *v*. medium, 8–12 years *v*. high, 13 or more years of schooling), marital status (married or cohabiting *v*. single or divorced or widowed), parity (number of births), smoking status (smoker *v*. non-smoker) and nutritional status (defined as underweight (BMI < 18·5 kg/m^2^), normal (BMI 18·5 to <25 kg/m^2^), overweight (BMI 25 to <30 kg/m^2^) and obese (BMI ≥ 30 kg/m^2^))^(33,34)^. BMI (kg/m^2^) was derived from height and weight.

### Statistical methods

Prevalence rates, means and medians were calculated with multistage sampling and adjusted following a post-stratification procedure using Chile’s 2017 census estimates. se and 95 % CI were calculated using the Taylor series expansion method^(35)^.

Analyses were based on complete cases. *P*-values <0·05 were classed as statistically significant (two-tailed). Associations between serum folate levels and all variables included in the study (socio-demographic, lifestyle and nutritional status) were established by univariate and multivariate linear regression analyses, using SPSS software (version 17.0). The multivariate linear regression model for serum folate levels was adjusted for age (years) and nutritional status (BMI (kg/m^2^) (continuous); educational level, marital status and parity (categorical); physical activity during leisure, current smoking status and folic acid supplementation (dichotomic). Median and percentiles were calculated using SAS 9.4 (2013) statistical software. All analyses were adjusted for the complex survey design of the ENS.

## Results

### Socio-demographic characteristics

The socio-demographic characteristics of the weighted sample are shown in Table 1. The current study involved 222 women between the ages of 15 and 49 years (mean age 31·2 ± 0·9 years old). Almost 91 % of women had medium or high educational levels (more than 8 years of schooling). Regarding their reproductive history, around one-third of the sample was nulliparous, one-third had given birth once or twice and the remainder had given birth more than three times. Thirty-two percentage of the women declared they were smokers. Eighty-eight percentage were classified as sedentary, and almost 70 % of the participants were either overweight or obese (mean BMI 27·9 ± 0·5 kg/m^2^). Only 1·2 % of the participants were taking folic acid supplements.

**Table 1:** Socio-demographic characteristics of women aged 15–49 years living in urban areas of the Metropolitan Region of Chile, Encuesta Nacional de Salud 2016–2017

	No. of participants	Expanded frequency (%)
Age range (years)		
15–19	27	13·9
20–29	74	33·8
30–39	71	28·9
40–49	50	23·3
Educational level		
Low (< 8 years of schooling)	12	4·7
Medium (8–12 years of schooling)	113	57·0
High (>12 years of schooling)	97	38·4
Marital status		
Married	93	35·4
Separated, divorced or widowed	20	10·4
Single	109	54·2
Parity (no. of pregnancies)*		
0	71	34·0
Between 1 and 2	78	34·2
3 or more	69	31·8
Smoking status		
Non-smoker	144	67·7
Smoker	78	32·3
Physical activity (during leisure time)		
No	196	88·0
Yes	26	12·0
Nutritional status		
Underweight	2	1·7
Normal	68	29·7
Overweight	86	40·9
Obese	66	27·7
Folic acid supplementation		
No	218	98·8 %
Yes	4	1·2 %

* Data from parity of four women were not reported.

### Plasma folate levels

Results indicating the prevalence of folate deficiency and excess are shown in Table 2. Only 0·9 % of the women presented serum folate deficiency with levels below 4·4 ng/ml. Most participants (92·1 %) showed serum folate levels that were in the physiological range (4·4–20 ng/ml). On the other hand, 7·0 % of women exhibited supraphysiological serum folate concentrations. In this group, 3·7 % exhibited high folate levels (>20 ng/ml), and 3·3 % were in the very high or highest folate (>26 ng/ml) category.

**Table 2:** Serum folate levels in women of childbearing age living in urban areas of the Metropolitan Region of Chile, Encuesta Nacional de Salud 2016–2017

Folate status (folate range in ng/ml)	Unweighted sample size (no. of participants)	Expanded frequency (%)
Deficient (<4·4)	2	0·9
Normal (4·4–20)	207	92·1
High (>20–26)	6	3·7
Very high (>26)	10	3·3

The mean serum folate level of women of childbearing age living in the urban MR was 14·2 ± 0·4 ng/ml, and the median was 13·5 ng/ml (range 2·1–32·2 ng/ml). Table 3 shows the percentile distribution in those women. Mean and median serum folate concentrations were similar across all socio-demographic variables measured (Table 4). A significant difference in serum folate levels was only found by folic acid supplementation (*P* = 0·028), where women with supplementation had, on average, 70 % higher levels of serum folate than women without folic acid supplementation (24·0 ± 5·2 *v*. 14·01 ± 0·4 ng/ml, respectively).

**Table 3:** Percentile distribution of serum folate in women of childbearing age living in urban areas of the Metropolitan Region of Chile

Percentile	5 %	10 %	20 %	30 %	40 %	50 %	60 %	70 %	80 %	90 %	95 %
Serum folate (ng/ml)	7·6	9·4	10·9	12·2	12·8	13·5	14·6	15·4	16·7	18·9	24·1
se	1·7	0·9	0·4	0·4	0·3	0·4	0·4	0·3	0·6	1·8	2·2

**Table 4:** Serum folate levels by socio-demographic characteristics in women of childbearing age living in urban areas of the Metropolitan Region of Chile

	No. of participants	Mean (ng/ml)	se (ng/ml)	Median (ng/ml)	Range (ng/ml)	*P*
Age range (years)						
15–19	27	13·6	1·0	13·0	2·1–27·4	0·57
20–29	74	14·3	0·9	13·8	4·7–32·2	
30–39	71	13·8	0·4	13·1	8·2–31·1	
40–49	50	15·0	0·8	14·3	2·1–27·9	
Educational level						
Low (≤8 years of schooling)	12	14·5	1·9	11·3	2·1–27·1	0·76
Medium (8–12 years of schooling)	113	13·9	0·5	13·4	2·1–31·1	
High (≥12 years of schooling)	97	14·6	0·7	13·9	5·7–32·2	
Marital status						
Married	93	14·1	0·6	13·4	2·1–31·1	0·90
Separated, divorced or widowed	20	13·9	0·8	14·4	5·7–24·8	
Single	109	14·4	0·6	13·5	2·1–32·2	
Parity (no. of pregnancies)						
0	71	14·7	0·6	13·5	5·5–32·2	0·64
Between 1 and 2	78	13·8	0·8	13·2	2·1–29·8	
3 or more	69	14·11	0·62	13·9	2·1–31·1	
Smoking status						
Non-smoker	144	14·1	0·5	13·4	2·1–31·1	0·68
Smoker	78	14·4	0·7	13·9	2·1–32·2	
Physical activity (during leisure time)						
No	196	14·2	0·4	13·4	2·1–32·2	0·70
Yes	26	14·4	0·5	14·7	4·7–27·4	
Nutritional status						
Underweight or normal	70	14·0	0·8	13·7	2·1–29·8	0·73
Overweight or obese	152	14·3	0·5	13·4	2·1–32·2	
Folic acid supplementation						
No	218	14·1	0·4	13·5	2·1–32·2	0·03
Yes	4	24·0	5·2	29·1	15·7–29·5	

Linear regression was performed to examine the relationship between serum folate concentrations with age, educational level, marital status, parity, smoking status, nutritional status and folic acid supplement use (Table 5). The only variable that showed a significant association with folate levels after adjusting for other variables was folic acid supplement use. Women who did not use folic acid supplements presented 9·9 ng/ml lower mean serum folate levels than those taking supplements. However, only 1·2 % of women in our study used supplements.

**Table 5:** Adjusted linear regression model for associations between serum folate levels with socio-demographic variables and nutritional status in women of childbearing age living in urban areas of the Metropolitan Region of Chile

Variable	*β*	95 % CI		*P*
		Lower limit	Upper limit	
Age (years)	0·053	−0·040	0·147	0·264
Educational level				0·939
Low	0·050	−3·366	4·465	0·782
Medium	−0·104	−1·851	1·644	0·907
High	0·000			
Marital status				0·897
Married	0·055	−2·075	2·186	0·959
Separated, divorced or widowed	−0·491	−3·274	2·291	0·728
Single	0·000			
Parity (no. of pregnancies)				0·488
0	0·790	−2·109	3·689	0·592
Between 1 and 2	−0·814	−2·678	1·050	0·390
3 or more	0·000			
Nutritional status				0·359
BMI (kg/m^2^)	−0·067	−0·211	0·077	0·359
Physical activity (during leisure time)				0·755
Yes	−0·221	−1·748	1·306	0·775
No	0·000			
Smoking status				0·564
No	−0·495	−2·187	1·196	0·564
Yes	0·000			
Folic acid supplementation				0·021
No	−10·893	−20 127	−1660	0·021
Yes	0·000			

Multivariate linear regression model for serum folate levels adjusted for age (years, continuous), educational level (low, medium and high), marital status (married; separated, divorced or widowed; single), parity (0, 1–2, ≥3 pregnancies), nutritional status (BMI (kg/m^2^), continuous), physical activity during leisure time (yes, no), smoking status (non-smoker, current smoker) and folic acid supplementation (yes, no).

## Discussion

In 2000, the implementation of mandatory folic acid fortification of wheat flour in Chile increased circulating folate levels in women of childbearing age^(14)^ and concomitantly reduced the incidence of NTD by 43 %^(15)^. The folate status of women of reproductive age in Chile was last analysed a few years after, in 2003, in women of low socio-economic households^(14)^.

The aim of this paper was to determine the current folate status in Chilean women of reproductive age belonging to different socio-demographic groups for the first time after the last adjustment to the folic acid fortification policy in 2009. The results show that folate levels were mostly adequate in women included in the current study. This information, together with current data on NTD prevalence in Chile, suggests that the current folic acid fortification policy is effective and equitable.

The mean serum folate concentration in Chilean women of reproductive age from the MR of Chile was 14·2 ng/ml, lower than the 16·3 ng/ml determined in Chilean women in 2003^(14)^. Serum folate levels were adequate in 89·1 % of women, and no disparities were detected according to socio-demographic variables previously shown to affect folate status, such as educational level, smoking status or BMI^(36)^. As expected, folic acid supplement intake was associated with higher serum folate levels, but only 1·2 % of the population took supplements. Despite this low level of supplement use, 7 % of women (including supplement users and non-users) exhibited supraphysiological serum folate levels.

Folate concentrations detected by the ENS 2016–2017 were lower to those in the ENS 2009–2010: the mean shifted from 21·2 to 14·2 ng/ml, and the p95 from 38·6 to 24·1 ng/ml. However, a direct comparison between serum folate levels from both surveys is inadequate because different laboratory techniques were used and different subpopulations were included in the ENS 2009–2010 and ENS 2016–2017. The former used direct competitive chemiluminescence immunoassays and included elderly participants, and the latter used competitive electrochemiluminescence-based immunoassay and analysed women of reproductive age, respectively. Indeed, existing evidence shows that higher levels of serum folate are usually detected in participants older than 60 years due to higher intake and/or lower catabolism of folic acid^(37)^. This difference may explain the lower level in serum folate in 2016–2017 *v*. 2009–2010 surveys. Future evaluation of folate status in Chileans will require the analysis of the target population (young women) in parallel to other populations potentially affected by the fortification policy, for example, children and elderly.

The WHO recommends the microbiological assay as the most reliable method to obtain comparable estimates of population folate status across different countries^(38)^. However, this method requires standardisation and adjustment of threshold levels to obtain reproducible and comparable results within and among laboratories^(38)^. In our country, the microbiological assay method was not available when the ENS 2016–2017 was undertaken, so we used electrochemiluminescence immunoassay, a commercial protein-based immunoassay used in clinical and research settings, to determine serum folate levels.

The current study shows that serum folate levels are mostly within the normal range in young women living in urban areas of the MR of Chile and suggests that the current folic acid fortification policy is adequate to keep NTD within folate-preventable ranges. In a recent publication, the mean reported NTD prevalence in Chile between 2001 and 2013 was 5·5:10 000^(13)^. The prevalence of NTD in 2018 was 6·4: 10 000 live births (C Mellado and RA Pardo, unpublished results). These proportions are in the range of 4–9:10 000 live births expected in countries that have reached adequate folate status^(38,39)^.

Our study has several limitations. Firstly, we were unable to determine folate insufficiency on the basis of an increased risk of NTD because of a mismatch between the assay used to establish this folate insufficiency cut-off (microbiological assay) and the assay we used to determine folate levels in our study (electrochemiluminescence immunoassay)^(38)^. Secondly, the current study only measured the folate levels in women of childbearing age living in the urban MR of Chile because folate integrity could not be ensured for samples transported to the clinical laboratory from other regions. Hence, our study is only representative of women residing in the MR, not in Chile as a whole. Nevertheless, it is important to note that approximately 40 % of Chileans live in the MR. Thirdly, we measured serum folate instead of erythrocyte folate, the latter being a better indicator of long-term folate levels^(36)^. Finally, folate intake was not assessed in the ENS by means of dietary questionnaires (e.g., 24-h recall, food diary or FFQ). This would have enabled us to verify if women of childbearing age are meeting folate intake recommendations. Furthermore, the correlation between serum biomarker levels and women’s reported intake might have served as an indirect measure of flour manufacturers’ compliance with regulations regarding folic acid fortification in Chile.

Our analysis highlights the importance of performing additional research in order to monitor folate status in different age and sex population subgroups at a national, using the gold standard methods for measuring long-term folate status (microbiological assay in erythrocytes), before considering any further adjustments to the national policy. Technical calculations of the burden of disease due to deficit *v*. excess of folate may also be required to assess the benefit *v*. risk of folic acid fortification in our country objectively. Also, it is fundamental to ensure strict surveillance of the flour and food industries to prevent excessive fortification and addition with folic acid.

Altogether, the results and discussion described above support maintaining the current fortification policy with folic acid as a measure to maintain folate-preventable ranges of NTD in Chile. The low awareness/adherence and socio-economic disparities in the use of vitamin supplements^(8,9,39,40)^ and the high proportion of unplanned pregnancies in our region^(4)^ further support this view.
